# User satisfaction in relation to Primary Health Care services in Brazil

**DOI:** 10.11606/s1518-8787.2021055002533

**Published:** 2021-05-06

**Authors:** Juliana Leal Ribeiro Cantalino, Magda Duarte dos Anjos Scherer, Jacks Soratto, Antônio Augusto Schäfer, Davllyn Santos Oliveira dos Anjos

**Affiliations:** I Universidade de Brasília Programa de Pós-Graduação em Saúde Coletiva Faculdade de Ciências da Saúde BrasíliaDF Brasil Universidade de Brasília. Faculdade de Ciências da Saúde. Programa de Pós-Graduação em Saúde Coletiva. Brasília, DF, Brasil.; II Universidade do Extremo Sul Catarinense Programa de Pós-Graduação em Saúde Coletiva CriciúmaSC Brasil Universidade do Extremo Sul Catarinense. Programa de Pós-Graduação em Saúde Coletiva. Criciúma, SC, Brasil.

**Keywords:** Primary Health Care, Patient Satisfaction, Health Evaluation, Health Services

## Abstract

**OBJECTIVE:**

To analyze user satisfaction in relation to access, infrastructure and quality of Primary Health Care (PHC) services in Brazil.

**METHODS:**

This cross-sectional study was conducted with data from 114,615 users linked to 30,523 health teams, obtained through the database of the *Programa Nacional de Melhoria do Acesso e da Qualidade da Atenção Básica* (PMAQ-AB —National Program for the Improvement of Access and Quality of Primary Care). Independent variables related to access, infrastructure and quality of services in PHC were studied. The outcome, user satisfaction, was measured using the variables: “if given the option, I would change the staff or health service” and “I would recommend this health service to a friend or family member.” To assess satisfaction according to independent exposure variables, Pearson’s chi-squared test was used, considering a significance level of 5%. Descriptive analyses of the variables were performed using absolute (n) and relative (%) frequencies.

**RESULTS:**

User satisfaction was associated with the variables of access (p < 0.001), infrastructure (p < 0.001) and quality of services (p < 0.001) in PHC. The proximity of the service, attention to spontaneous demand, listening and the respect of professionals to the singularities of the patient, as well as the problem-solving capacity of the services, without the need for referrals to others and the good infrastructure, were related to user satisfaction.

**CONCLUSION:**

To ensure the improvement of the quality of services offered in PHC in Brazil, the aspects of user satisfaction identified in this study should be considered in the organization and management of services.

## INTRODUCTION

User satisfaction with services should be considered in the assessment and planning of Primary Health Care (PHC) actions. Satisfaction permeates several factors, including ensuring access to services when necessary, the quality of care provided by professionals and the structural conditions of the places that provide assistance^1^^,^^2^. User satisfaction is something complex and difficult to assess because it has a subjective dimension and a strong relationship with the characteristics of the work process.

PHC is an important international strategy for universal health systems. In Brazil, it was materialized by a series of individual or collective health actions of promotion, prevention, diagnosis, harm reduction and health surveillance, carried out by multidisciplinary teams working in adstrict territory^3^.

The expansion of PHC in recent years, with population coverage of around 74% in the national territory^4^, has contributed to the reduction in morbidity and mortality indicators, with significant contributions to reducing infant mortality and hospitalizations for causes sensitive to primary care^5^. However, access is still very heterogeneous, sometimes being precarious^6^,causing the dissatisfaction of users^7^ and indicating a situation that challenges professionals, managers and researchers to rethink actions for its improvement. Access is the ability of PHC to welcome users by promoting the connection with professionals and co-responsibility for the care of their health needs. Acting on the perspective of improving access means contributing to the quality of health care^5^.

The quality of services has been the target of complaints from users, since the delay in scheduling appointments and the non-attendance to the continuous demand that arises in the services are the main factors for user dissatisfaction with PHC^8^. Also, PHC infrastructure is another aspect that deserves attention, after all, the structure of services must consider the precepts of ambience, in order to provide a welcoming and humane attention to users, in addition to a comfortable environment for the work of health professionals^3^. Even with financial incentive from the Ministry of Health for the requalification of health services^9^, there is still much to advance, considering that many still present precarious situations also for professionals, with weaknesses in the physical, organizational structure and lack of materials and inputs^10^^–^^13^.

In view of the above, this study aims to analyze the user satisfaction in relation to access, infrastructure and quality of services in PHC in Brazil.

## METHODS

This is a cross-sectional study with secondary data obtained through the national database of the external assessment of the 2nd Cycle of the *Ciclo do Programa Nacional de Melhoria do Acesso e da Qualidade da Atenção Básica* (PMAQ-AB — National Program for The Improvement of Access and Quality of Primary Care), conducted in 2014^14^. The PMAQ-AB is a performance compensation program of the Ministry of Health whose main objective is to encourage the expansion of access and improvement of the quality of primary care, with the aim of establishing a comparable quality standard nationally, regionally and locally. Through tools for assessing and making available the collected data, the PMAQ-AB enables transparency and effectiveness of government actions in this field of operation^14^.

The Program was designed in four complementary phases: 1) program adhering; 2) implementation of reorganization devices of team and management work processes (self-assessment, continuing education, institutional support and monitoring); 3) external assessment, made by specialists and users; 4) agreement with increasing standards and quality indicators^14^.

For external evaluation, six instruments with different objectives were applied, and in this study, variables of module 3 were used, which refers to the interview to assess user satisfaction with primary care services offered by Basic Health Units (BHU)^15^.

The application of module 3 occurred through a structured interview that sought to know the sociodemographic characteristics of the participants and the satisfaction in relation to the services offered. Users were randomly selected, four by BHU assessed, with the following inclusion criteria: users who did not have an appointment with a doctor, nurse or dentist on the day of the interview; over 18 years of age; who were not conducting the first appointment; and who were not returning after absence of more than 12 months in relation to the date of the interview. When the selected user did not meet the criteria, another user was randomly selected, and no refusals were recorded.

We emphasize that the PMAQ is conducted in a partnership between the Ministry of Health and 41 teaching and research institutions, which conducted exhaustive training with the nucleus of interviewers and supervisors responsible for data collection to provide a national standardization of the data in order to improve the quality of records. The data collection instrument, because it was performed via an application installed on tablets, had a set of validation rules in its programming, which performed the internal validation of the answers while the instrument was filled. In a third stage, the data were validated by the Ministry of Health system to assess the completeness of the questionnaires and the identification data of the interviewed teams. Only after these steps were the teams certified, and the databases were made available for the public access of the researchers.

The expected population was 122,092 users, however, there were 7,477 (6.12%) losses due to the absence of users in PHC services to answer the questionnaire at the time of data collection or because they were from teams that gave up being assessed. In the end, 114,615 users of the 30,523 primary care health teams in Brazil were included, covering 5,057 (90.8%) municipalities in Brazil that were part of the 2nd cycle of the PMAQ-AB.

For this study, 7.2% of the 319 variables in module 3 were selected by the researchers, totaling 23 variables. Of these, 17 are independent variables related to access, infrastructure and quality of services in PHC (described in Tables 2, 3 and 4). For analysis purposes, these variables were dichotomized when they presented more than two response categories. The outcome, user satisfaction, was measured using two variables: “if given the option, I would change the team or health service” (yes/no) and “I would recommend this health service to a friend or family member” (yes/no). Sociodemographic variables were also included: gender (male, female), age group (≤ 29, 30–54, 55–64, ≥ 65), skin color (white, black, brown/mixed race, yellow, indigenous), schooling (not literate [cannot read and write], is literate [knows how to read and write], some elementary school, elementary school, some high school, high school, some higher education, higher education), in order to characterize the population.

Data analysis was performed using the statistical software IBM SPSS version 20.0. Pearson’s Chi-square test was used to assess user satisfaction according to access, infrastructure and quality of services in PHC, pearson’s Chi-square test was used, considering a significance level of 5%. Descriptive analyses of the variables were performed using absolute (n) and relative (%) frequencies.

## RESULTS

Most respondents were women (79.6%), predominantly the age group between 30 and 54 years (44.4%), race/skin color self-declared brown/mixed race (46.2%) and with some elementary school (36.0%) (Table 1).

**Table 1:** Sociodemographic characteristics of the users studied. Brazil, 2014 (n = 114,615).

Characteristics		n	%
Gender			
	Male	23,412	20.4
	Female	91,203	79.6
Age group (years)			
	≤ 29	27,479	24.0
	30–54	50,899	44.4
	55–64	18,771	16.4
	≥ 65	17,466	15.2
Race/skin color (n = 112,472)ᵃ			
	White	41,145	36.6
	Black	15,010	13.3
	Brown/mixed	51,909	46.2
	Yellow	3,528	3.1
	Indigenous	880	0.8
Schooling			
	Not literate (cannot read and write)	8,669	7.6
	Literate (can read and write)	8,491	7.4
	Some elementary school	41,213	36.0
	Elementary school	12,169	10.6
	Some high school	11,966	10.5
	High school	24,840	21.7
	Some higher education	3,078	2.7
	Higher education	4,045	3.5

a Maximum percentage of unknown observations for the variable race/skin color: 2,143 (1.9%).

Table 2 shows the relationship between access and user satisfaction. We observed that individuals who would recommend the health service to a friend or family member and who would not change team or health service are those who answered that the time of travel from their residence to the health service was less than 30 minutes (p < 0.001), who considered access to the health service very easy or easy (p < 0.001), who reported that the opening hours of the health service meet their needs (p < 0.001) and they considered the quality of care to be very good or good when there is no scheduled time (p < 0.001).

**Table 2:** User satisfaction in relation to access to primary health care services in Brazil. Brazil, 2014 (n = 114,615).

		total n	If I had the option, I would change staff or health service					I would recommend the health service to a friend or family member				
			Yes		No		p-valor	Yes		No		p
			n	%	n	%		n	%	n	%	
Time of commuting from home to the Health Service		114,195										
	< 30 minutes		15,072	16.0	79,398	84.0	< 0.001	81,973	86.8	12,497	13.2	< 0.001
	≥ 30 minutes		5,093	25,8	14,632	74.2		16,297	82.6	3,428	17.4	
Easy access to the Health Service		114,615										
	Very easy/easy		13,568	14.7	78,523	85.3	< 0.001	80,541	87.5	11,550	12.5	< 0.001
	Reasonable/difficult and very difficult		6,668	29.6	15,856	70.4		18,076	80.3	4,448	19.7	
Opening hours of the health service meet the needs		113,076										
	Yes		13,374	13.6	84,824	86.4	< 0.001	87,976	89.6	10,222	10.4	< 0.001
	No		6,450	43.4	8,428	56.6		9,509	63.9	5,369	36.1	
Quality of service when there is no scheduled time		67,277										
	Very good/good		6,717	12.1	48,983	87.9	< 0.001	50,810	91.2	4,890	8.8	< 0.001
	Regular/bad/very bad		5,723	49.4	5,854	50.6		7,225	62.4	4,352	37.6	

Regarding the quality of care and the satisfaction of the users studied (Table 3), the results indicate that individuals who would recommend the health service to a friend or family member and who would not change teams or health services were those who considered that always – or most of the time – the team sought to solve their needs/problems in the health service itself (p < 0.001). In addition, they also considered that the medical office is a reserved place, which provides privacy during the appointment (p < 0.001), that always – or most of the time – professionals provide guidance on the need for rest, adequate nutrition and how to take medicines (p < 0.001), as well as feel respected by professionals in relation to cultural habits, customs and religion (p < 0.001). They also considered that the time of appointment with the doctor and nurse is sufficient (p < 0.001), who always – or most of the time – have the facility to talk to the professionals who treat them to ask questions after appointments (p < 0.001), in the same way they find it easy to talk to professionals about the results of the exams (p < 0.001) and referred to the quality of care received by the health team as very good and good (p < 0.001).

**Table 3:** User satisfaction in relation to the quality of primary health care services. Brazil, 2014 (n = 114,615).

		total n	If I had the option, I would change staff or health service					I would recommend the health service to a friend or family member				
			Yes		No		p	Yes		no		p
			total n	%	n	%		total n	%	n	%	
The team seeks to solve the needs/problems in the Health Service itself		113,185										
	Always/most of the time		13,887	13.7	87,799	86.3	< 0,001	91,122	89.6	10,564	10,4	< 0.001
	Hardly ever/never		6,037	52.5	5,462	47.5		6,362	55.3	5,137	44,7	
Medical office is a reserved and private place	113,859										
	Yes		17,338	16.1	90,375	83.9	< 0,001	94,181	87.4	13,532	12,6	< 0.001
	No		2,713	44.1	3,433	55.9		3,868	62.9	2,278	37,1	
Professionals provide guidance on the need for rest, adequate nutrition and how to take medicines		113,409										
	Always/most of the time		14,864	14.7	86,072	85.3	< 0,001	89,614	88.8	11,322	11,2	< 0.001
	Hardly ever/never		5,114	41.0	7,359	59.0		8,046	64.5	4,427	35,5	
Feels respected by professionals in relation to cultural habits, customs, religion		113,777										
	Always/most of the time		17,905	16.3	92,057	83.7	< 0,001	96,133	87.4	13,829	12,6	< 0.001
	Hardly ever/never		2079	54.5	1,736	45.5		1,896	49.7	1,919	50,3	
Appointment with the doctor is sufficient		112,372										
	Yes		11,864	12.7	81,275	87.3	<0,001	83,616	89.8	9,523	10,2	< 0.001
	No		7,854	40.8	11,379	59.2		13,230	68.8	6,003	31,2	
Appointment with the nurse is sufficient		103,789										
	Yes		13,525	14.3	81,281	85.7	< 0,001	84,159	88.8	10,647	11,2	< 0.001
	No		4,082	45.4	4,901	54.6		5,689	63.3	3,294	36,7	
It is easy to talk to the professionals who treat you to ask questions after appointments		79,216										
	Always/most of the time		8,062	12.3	57,404	87.7	< 0,001	59,452	90.8	6,014	9,2	< 0.001
	Hardly ever/never		6,073	44.2	7,677	55.8		8,766	63.8	4,984	36,2	
Find it easy to talk to professionals about exam results		105,586										
	Always/most of the time		11,089	13.0	74,363	87.0	< 0,001	76,967	90.1	8,485	9,9	< 0.001
	Hardly ever/never		7,424	36.9	12,710	63.1		14,022	69.6	6,112	30,4	
Quality of care received by the health team		114,378										
	Very good/good		9,381	10.0	84,876	90.0	< 0,001	87,284	92.6	6,973	7,4	< 0.001
	Regular/bad/very bad		10,780	53.6	9,341	46.4		11,205	55.7	8,916	44,3	

As to the association between infrastructure and user satisfaction (Table 4), individuals who would recommend the health service to a friend or family member and who would not change staff or service were those who reported: good conditions of use and cleaning of health service facilities (p < 0.001); sufficient number of chairs for people to sit in the waiting place (p < 0.001); very good or good perception about health service facilities (p < 0.001).

**Table 4:** User satisfaction in relation to the infrastructure of primary health care services. Brazil, 2014 (n = 114,615).

		total n	If I had the option, I would change staff or health service					I would recommend the health service to a friend or family member				
			Yes		No		p	Yes		No		p
			n	%	n	%		n	%	n	%	
Health service facilities are in good condition for use		114,615										
	Yes		10,164	11.9	75,038	88.1	< 0,001	77,241	90.7	7,961	9.3	< 0,001
	No		10,072	34.2	19,341	65.8		21,376	72.7	8,037	27.3	
The facilities of the health service are in good cleaning condition		114,615										
	Yes		13,526	13.8	84,460	86.2	< 0,001	87,562	89.4	10,424	10.6	< 0,001
	No		6,710	40.4	9,919	59.6		11,055	66.5	5,574	33.5	
Enough chairs for people to sit in the waiting place		114,615										
	Yes		6,265	10.2	55,390	89.8	< 0,001	56,449	91.6	5,206	8.4	< 0,001
	No		13,971	26.4	38,989	73.6		42,168	79.6	10,792	20.4	
Perception of health service facilities		114,071										
	Very good/good		6,278	8.6	66,958	91.4	< 0,001	67,891	92.7	5,345	7.3	< 0,001
	Regular/bad/very bad		13,860	33.9	26,975	66.1		30,297	74.2	10,538	25.8	

## DISCUSSION

An important finding of this study is that most users interviewed reported satisfaction regarding access, infrastructure and quality of services in PHC in Brazil.

The distance between the health service and the household played a significant role in satisfaction, corroborating the results of the evaluation of the 1st Cycle of the PMAQ-AB^16^. The satisfaction may be related to the possibility of not using means of transportation to arrive at the service and with the short time required to carry out the route^17^. Analysis of data from the 2012 PMAQ-AB in Pernambuco showed that most users (73.2%) considered that health services were close to their homes, something already expected, considering that health services with less complexity tend to be closer to the population^18^. However, the simple proximity, however important an aspect to influence user satisfaction, does not guarantee the real use.

As to user satisfaction regarding working hours, several studies carried out within the scope of PHC, corroborating this study, found positive results^17^^–^^19^. The operation of the PHC service in at least five days a week, with a workload of at least eight hours, with availability of medications and test results, as well as appointments with assured specialists, in addition to a greater number of health units in the municipality, were identified as factors that make the service more accessible and, thus, satisfactory for users^17^^–^^19^.

However, the results of the research on the 1st cycle, conducted between 2012 and 2013, showed that the functioning of PHC, only during business hours, does not meet the needs of the population interviewed in the five major Brazilian regions^16^. The closure of PHC services for a certain period for the meetings of the teams, recommended as essential by the national guidelines for work in PHC, contributes to the user dissatisfaction regarding the hours of operation of the services^17^.

Another important item of user satisfaction in relation to access is the fulfillment of spontaneous demand. We observed that, between cycles 1 and 2 of the PMAQ-AB, the reservations of vacancies for same-day care went from 64.9% to 88.1%, signaling improvement in this indicator^20^. Of the users seeking the PHC services for some immediate call, most report having obtained the appointment^19^.

The results found regarding the ease of access to services by most users interviewed coincide with that found in the assessment of the 1st cycle of the PMAQ-AB, in which 83% of PHC teams were classified as accessible to users^19^. Technical-care, economic, political and symbolic conditions are constituents of the concept of health services accessibility^7^, however, these conditions can be affected by issues such as location, population and socioeconomic characteristics, which can influence access to PHC services^21^. Some attributes of PHC showed improvement between PMAQ cycles 1 and 2, such as integrality and first contact^20^. These attributes may have influenced the level of user satisfaction with the service and contributed to reduce inequalities in the access and use of health services in Brazil^22^.

As to the high percentage of users who considered that health professionals and services are accessible and resolutive to their needs when seeking PHC services, this positive outcome may have been produced by the fragility in the selection process of the interviewees, since it only considered the opinion of the group that obtained access to care, which is one of the limiting factors of this study.

The weaknesses in the PHC care coordination network influence the percentage of problem-solving capacity of users’ health problems^23^. If the correct understanding of the user’s need can be performed and coordinated by primary care, the gateway to the Unified Health System (SUS), the percentage of satisfaction regarding the service can be increased, which leads them to indicate PHC services to their family members and acquaintances^24^. This may happen because the user does not have to go a long way in the specialized network or high complexity.

Regarding the existence of an office for patient privacy, the results found confirm studies that identified that user satisfaction decreases by 41% when the office is not in a reserved area^25^, either for changing clothes or performing exams^26^. The lack of privacy has an ethical dimension, affects human dignity, hinders listening, the development of bonding and trust in the relationship between user and professional. These aspects are related to the time the medical professional or nurse spends with the patient, which is also significantly associated with user satisfaction^1^^,^^27^^,^^28^.

As for cultural habits, customs and religion, individuals who feel respected by professionals have a strong influence on the positive assessment of health services^1^^,^^25^, as evidenced by this study and Protasio et al.^25^ This aspect of satisfaction may be associated with the production of modes of care, which when guided by the “valorization of knowledge, experience and autonomy of users, by the understanding of the other as being similar, tend to contribute to more creative actions that qualify care in PHC”^29^.

In relation to the infrastructure of PHC services, we found a predominance of positive assessment of the facilities by users. Political, organizational and structural conditions that ensure the execution of essential functions of health units is a condition for greater user satisfaction and a challenge to build a strong and comprehensive PHC^30^.

The study showed that user satisfaction is associated with elementary aspects for the functioning of PHC services, which depend on the commitment of managers to the spaces of care, which must have good physical facilities, healthy hygiene and cleaning conditions, furniture, materials and enough supplies for the exercise of professional practices.

The reduction in user satisfaction to the dimensions of access, quality of care and infrastructure of services, as well as the difficulty in conceptualizing the term quality in the assessment of a service, are some of the limitations to this study. However, the variables selected to assess satisfaction allowed us to outline an overview of how users perceive PHC services. The use of secondary data, in general, could constitute a limitation to the study; however, the secondary data used in this study are from a public database from a national research that followed the criteria of standardization of collection in order to provide quality of records.

The elements of user satisfaction regarding access, quality and infrastructure of health services confirm the importance of ensuring, increasingly, that people are able not only to access health services, but also be treated in places with adequate physical structures and with professionals able to deal with differences and singularities.

Users recommend and would not change team or health service when there is a short time of commuting to the service, easy access, good working hours, quality of care to spontaneous demand, resolution of problems in the service itself, existence of health guidelines, respect for diversities, sufficient time for the appointment, ease to solve various doubts about exams, quality of care received, and good infrastructure.

Given this perspective, to ensure the improvement of the quality of services offered in PHC in Brazil, the aspects of user satisfaction identified in this study should be considered in the organization and management of services.
