# Supplementary material for: User satisfaction in relation to Primary Health Care services in Brazil
Source: Rev Saude Publica. 2021 May 6;55:22. doi: 10.11606/s1518-8787.2021055002533 (PMC8102024; doi:10.11606/s1518-8787.2021055002533)
Supplement: Translation

# Translation: Satisfação dos usuários em relação aos serviços de Atenção Primária à Saúde no Brasil

**Authors:** Juliana Leal Ribeiro Cantalino, Magda Duarte dos Anjos Scherer, Jacks Soratto, Antônio Augusto Schäfer, Davllyn Santos Oliveira dos Anjos

**Affiliations:** I BrasíliaDF Brasil Universidade de Brasília. Faculdade de Ciências da Saúde. Programa de Pós-Graduação em Saúde Coletiva. Brasília, DF, Brasil.; II CriciúmaSC Brasil Universidade do Extremo Sul Catarinense. Programa de Pós-Graduação em Saúde Coletiva. Criciúma, SC, Brasil.

**Keywords:** Atenção Primária à Saúde, Satisfação do Paciente, Avaliação em Saúde, Serviços de Saúde

## Abstract

**OBJETIVO:**

Analisar a satisfação de usuários em relação ao acesso, à infraestrutura e à qualidade dos serviços na Atenção Primária à Saúde (APS) no Brasil.

**MÉTODOS:**

Estudo transversal com dados de 114.615 usuários vinculados a 30.523 equipes de saúde, obtidos por meio do banco de dados do Programa Nacional de Melhoria do Acesso e da Qualidade da Atenção Básica (PMAQ-AB). Foram estudadas variáveis independentes relacionadas ao acesso, à infraestrutura e à qualidade dos serviços na APS. O desfecho, satisfação dos usuários, foi mensurado através das variáveis: “se tivesse a opção, mudaria de equipe ou de serviço de saúde” e “recomendaria este serviço de saúde para um amigo ou familiar”. Para avaliar a satisfação de acordo com as variáveis independentes de exposição, foi utilizado o teste Qui-quadrado de Pearson, considerando nível de significância de 5%. Análises descritivas das variáveis foram realizadas através de frequências absoluta (n) e relativa (%).

**RESULTADOS:**

A satisfação dos usuários esteve associada às variáveis de acesso (p < 0,001), infraestrutura (p < 0,001) e qualidade dos serviços (p < 0,001) na APS. A proximidade do serviço, atenção à demanda espontânea, escuta e o respeito dos profissionais às singularidades do paciente, assim como a resolutividade dos serviços, sem necessidade de encaminhamentos para outros e a boa infraestrutura, estiveram relacionados com a satisfação dos usuários.

**CONCLUSÃO:**

Para a garantia da melhoria da qualidade dos serviços ofertados na APS do Brasil, os aspectos de satisfação dos usuários identificados no presente estudo devem ser considerados na organização e gestão dos serviços.

## INTRODUÇÃO

A satisfação dos usuários com os serviços deve ser considerada na avaliação e no planejamento das ações de Atenção Primária à Saúde (APS). A satisfação perpassa vários fatores, entre os quais se encontram a garantia de acesso aos serviços quando necessário, a qualidade do atendimento prestado pelos profissionais e as condições estruturais dos locais que concedem assistência^1^^,^^2^. A satisfação do usuário é algo complexo e difícil de avaliar pois tem dimensão subjetiva e forte relação com as características do processo de trabalho.

A APS constitui importante estratégia internacional dos sistemas universais de saúde. No Brasil, foi materializada por uma série de ações de saúde individuais ou coletivas de promoção, prevenção, diagnóstico, redução de danos e vigilância em saúde, realizada por equipes multiprofissionais que atuam em território adstrito^3^.

A expansão da APS nos últimos anos, com cobertura populacional em torno de 74% no território nacional^4^, tem colaborado para redução dos indicadores de morbimortalidade, com contribuições significativas na redução da mortalidade infantil e nas internações por causas sensíveis à atenção primária^5^. Entretanto, o acesso ainda é muito heterogêneo, sendo por vezes precário^6^, causando a insatisfação dos usuários^7^ e indicando uma situação que desafia profissionais, gestores e pesquisadores a repensarem ações para sua melhoria. O acesso é a capacidade da APS de acolher os usuários promovendo a vinculação com os profissionais e corresponsabilização pela atenção às suas necessidades de saúde. Atuar na perspectiva de melhora do acesso significa contribuir para qualidade da atenção à saúde^5^.

A qualidade dos serviços tem sido alvo de queixas dos usuários, uma vez que a demora para agendamento de consultas e a não assistência à demanda contínua que surge nos serviços constituem os principais fatores para insatisfação dos usuários com a APS^8^. Além disso, a infraestrutura da APS é outro aspecto que merece atenção, afinal, estrutura dos serviços deve levar em conta os preceitos da ambiência, de modo a proporcionar uma atenção acolhedora e humana para os usuários, além de um ambiente confortável para o trabalho dos profissionais de saúde^3^. Mesmo com incentivo financeiro do Ministério da Saúde para requalificação dos serviços de saúde^9^, ainda há muito a avançar, tendo em vista que muitos ainda apresentam situações precárias também para os profissionais, com fragilidades na estrutura física, organizacional e falta de materiais e insumos^10^^–^^13^.

Diante do exposto, o presente estudo tem por objetivo analisar a satisfação de usuários em relação ao acesso, à infraestrutura e à qualidade dos serviços na APS no Brasil.

## MÉTODOS

Trata-se de um estudo transversal com dados secundários obtidos por meio do banco de dados nacional da avaliação externa do 2º Ciclo do Programa Nacional de Melhoria do Acesso e da Qualidade da Atenção Básica (PMAQ-AB), realizado no ano de 2014^14^. O PMAQ-AB é um programa de remuneração por desempenho do Ministério da Saúde que tem como objetivo principal incentivar a ampliação do acesso e a melhoria da qualidade da atenção básica, como é denominada a atenção primária no Brasil, com o intuito de estabelecer um padrão de qualidade comparável nacional, regional e localmente. Através de ferramentas de avaliação e disponibilização dos dados coletados, o PMAQ-AB possibilita transparência e efetividade das ações governamentais nesse âmbito de atuação^14^.

O Programa foi concebido em quatro fases complementares: 1) adesão ao programa; 2) implantação de dispositivos de reorganização dos processos de trabalho da equipe e da gestão (autoavaliação, educação permanente, apoio institucional e monitoramento); 3) avaliação externa, feita por especialistas e por usuários; 4) pactuação com o incremento de padrões e indicadores de qualidade^14^.

Para avaliação externa foram aplicados seis instrumentos com objetivos diferentes, sendo que, neste estudo, foram utilizadas variáveis do módulo 3, que se refere à entrevista para avaliação da satisfação dos usuários com os serviços de Atenção Primária ofertados pelas Unidades Básicas de Saúde (UBS)^15^.

A aplicação do módulo 3 ocorreu por meio de entrevista estruturada que buscou conhecer as características sociodemográficas dos participantes e a satisfação em relação aos serviços ofertados. Os usuários foram selecionados de forma aleatória, sendo quatro por UBS avaliada, com os seguintes critérios de inclusão: usuários que não passaram por consulta com médico, enfermeiro ou dentista no dia da entrevista; com idade acima de 18 anos; que não estivessem realizando a primeira consulta; e que não estavam retornando depois de ausência de mais de 12 meses em relação à data da entrevista. Quando o usuário selecionado não atendia aos critérios, outro era selecionado aleatoriamente, não tendo sido registradas recusas.

Ressaltamos que o PMAQ é realizado numa parceria entre o Ministério da Saúde e 41 instituições de ensino e pesquisa, que realizaram treinamento exaustivo com o núcleo de entrevistadores e supervisores responsáveis pela coleta dos dados a fim de proporcionar uma padronização nacional dos dados com o objetivo de melhorar a qualidade dos registros. O instrumento de coleta de dados, por ser realizado via aplicativo instalado em *tablets*, possuía um conjunto de regras de validação em sua programação, que realizava a validação interna das respostas enquanto o instrumento era preenchido. Em uma terceira etapa, os dados eram validados pelo sistema do Ministério da Saúde com o objetivo de avaliar a completude dos questionários e os dados de identificação das equipes entrevistadas. Somente após essas etapas foi realizada a certificação das equipes e a disponibilização dos bancos de dados para o acesso público dos pesquisadores.

A população esperada era de 122.092 usuários, entretanto, foram registrados 7.477 (6,12%) perdas por ausência de usuário nos serviços de APS para responder ao questionário no momento da coleta de dados ou por serem de equipes que desistiram de serem avaliadas. Ao final, foram incluídos 114.615 usuários das 30.523 equipes de saúde da atenção primária do Brasil, abrangendo 5.057 (90,8%) municípios do Brasil que fizeram parte do 2º ciclo do PMAQ-AB.

Para o presente estudo, foram selecionadas pelos pesquisadores 7,2% das 319 variáveis do módulo 3, totalizando 23 variáveis. Dessas, 17 são variáveis independentes relacionadas ao acesso, à infraestrutura e à qualidade dos serviços na APS (descritas nas tabelas 2, 3 e 4). Para fins de análise, essas variáveis foram dicotomizadas quando apresentavam mais de duas categorias de resposta. O desfecho, satisfação dos usuários, foi mensurado através de duas variáveis: “se tivesse a opção, mudaria de equipe ou de serviço de saúde” (sim/não) e “recomendaria este serviço de saúde para um amigo ou familiar” (sim/não). Também foram incluídas as variáveis sociodemográficas: sexo (masculino, feminino), faixa etária (≤ 29, 30–54, 55–64, ≥ 65), cor da pele (branca, preta, parda/mestiça, amarela, indígena), escolaridade (não é alfabetizado [não sabe ler e escrever], é alfabetizado [sabe ler e escrever], ensino fundamental incompleto, ensino fundamental completo, ensino médio incompleto, ensino médio completo, ensino superior incompleto, ensino superior completo), a fim de caracterizar a população.

A análise dos dados foi realizada através do *software* estatístico IBM SPSS versão 20.0. Para avaliar a satisfação dos usuários de acordo com o acesso, a infraestrutura e a qualidade dos serviços na APS, foi utilizado o teste Qui-quadrado de Pearson, considerando nível de significância de 5%. Análises descritivas das variáveis foram realizadas através de frequências absoluta (n) e relativa (%).

## RESULTADOS

A maioria dos entrevistados era do sexo feminino (79,6%), predominando a faixa etária entre 30 e 54 anos (44,4%), raça/cor da pele autodeclarada parda/mestiça (46,2%) e com ensino fundamental incompleto (36,0%) (Tabela 1).

**Tabela 1:** Características sociodemográficas dos usuários estudados. Brasil, 2014 (n = 114.615).

Características		n	%
Sexo			
	Masculino	23.412	20,4
	Feminino	91.203	79,6
Faixa etária (anos)			
	≤ 29	27.479	24,0
	30–54	50.899	44,4
	55–64	18.771	16,4
	≥ 65	17.466	15,2
Raça/Cor da pele (n = 112.472)^a^			
	Branca	41.145	36,6
	Preta	15.010	13,3
	Parda/mestiça	51.909	46,2
	Amarela	3.528	3,1
	Indígena	880	0,8
Escolaridade			
	Não é alfabetizado (não sabe ler e escrever)	8.669	7,6
	É alfabetizado (sabe ler e escrever)	8.491	7,4
	Ensino fundamental incompleto	41.213	36,0
	Ensino fundamental completo	12.169	10,6
	Ensino médio incompleto	11.966	10,5
	Ensino médio completo	24.840	21,7
	Ensino superior incompleto	3.078	2,7
	Ensino superior completo	4.045	3,5

a Percentual máximo de observações desconhecidas para a variável raça/cor da pele: 2.143 (1,9%).

A Tabela 2 mostra a relação entre acesso e satisfação dos usuários. Pode-se observar que os indivíduos que recomendariam o serviço de saúde a um amigo ou familiar e que não mudariam de equipe ou serviço de saúde são aqueles que responderam que o tempo de deslocamento da sua residência ao serviço de saúde foi menor do que 30 minutos (p < 0,001), que consideraram muito fácil ou fácil o acesso ao serviço de saúde (p < 0,001), que referiram que o horário de funcionamento do serviço de saúde atende às suas necessidades (p < 0,001) e que consideraram muito boa ou boa a qualidade do atendimento quando não se tem horário agendado (p < 0,001).

**Tabela 2:** Satisfação de usuários em relação ao acesso a serviços de atenção primária à saúde. Brasil, 2014 (n = 114.615).

		n total	Se tivesse a opção, mudaria de equipe ou serviço de saúde					Recomendaria o serviço de saúde para um amigo ou familiar				
			Sim		Não		p-valor	Sim		Não		p
			n	%	n	%		n	%	n	%	
Tempo de deslocamento do domicílio ao Serviço de Saúde		114.195										
	< de 30 minutos		15.072	16,0	79.398	84,0	< 0,001	81.973	86,8	12.497	13,2	< 0,001
	≥ a 30 minutos		5.093	25,8	14.632	74,2		16.297	82,6	3.428	17,4	
Facilidade de acesso ao Serviço de Saúde		114.615										
	Muito fácil/fácil		13.568	14,7	78.523	85,3	< 0,001	80.541	87,5	11.550	12,5	< 0,001
	Razoável/difícil e muito difícil		6.668	29,6	15.856	70,4		18.076	80,3	4.448	19,7	
Horário de funcionamento do serviço de saúde atende às necessidades		113.076										
	Sim		13374	13,6	84.824	86,4	< 0,001	87.976	89,6	10.222	10,4	< 0,001
	Não		6450	43,4	8.428	56,6		9.509	63,9	5.369	36,1	
Qualidade do atendimento quando não tem horário agendado		67.277										
	Muito boa/boa		6717	12,1	48.983	87,9	< 0,001	50.810	91,2	4.890	8,8	< 0,001
	Regular/ruim/muito ruim		5723	49,4	5.854	50,6		7.225	62,4	4.352	37,6	

Quanto à qualidade do atendimento e à satisfação dos usuários estudados (Tabela 3), os resultados indicam que os indivíduos que recomendariam o serviço de saúde a um amigo ou familiar e que não mudariam de equipe ou serviço de saúde eram aqueles que consideraram que sempre – ou na maioria das vezes – a equipe buscava resolver as suas necessidades/problemas no próprio serviço de saúde (p < 0,001). Além disso, também consideraram que o consultório para atendimento é um lugar reservado, que proporciona privacidade durante a consulta (p < 0,001), que sempre – ou na maioria das vezes – os profissionais fornecem orientações sobre necessidade de repouso, alimentação adequada e como tomar os remédios (p < 0,001), assim como sentem-se respeitados(as) pelos profissionais em relação aos hábitos culturais, costumes e religião (p < 0,001). Consideraram também que o tempo de consulta com o médico e enfermeiro é suficiente (p < 0,001), que sempre – ou na maioria das vezes – têm facilidade para falar com os profissionais que lhes atenderam para tirar dúvidas após consultas (p < 0,001), da mesma forma que encontram facilidade para conversar com os profissionais sobre os resultados dos exames (p < 0,001) e se referiram à qualidade do cuidado recebido pela equipe de saúde como muito boa e boa (p < 0,001).

**Tabela 3:** Satisfação de usuários em relação à qualidade do atendimento em serviços de atenção primária à saúde. Brasil, 2014 (n = 114.615).

		n total	Se tivesse a opção, mudaria de equipe ou serviço de saúde					Recomendaria o serviço de saúde para um amigo ou familiar				
			Sim		Não		p	Sim		Não		p
			n	%	n	%		n	%	n	%	
A equipe busca resolver as necessidades/problemas no próprio Serviço de Saúde		113.185										
	Sempre/na maioria das vezes		13.887	13,7	87.799	86,3	< 0,001	91.122	89,6	10.564	10,4	< 0,001
	Quase nunca/nunca		6.037	52,5	5.462	47,5		6.362	55,3	5.137	44,7	
Consultório para atendimento é um lugar reservado, tem privacidade		113.859										
	Sim		17.338	16,1	90.375	83,9	< 0,001	94.181	87,4	13.532	12,6	< 0,001
	Não		2.713	44,1	3.433	55,9		3.868	62,9	2.278	37,1	
Os profissionais fornecem orientações sobre necessidade de repouso, alimentação adequada e como tomar os remédios		113.409										
	Sempre/na maioria das vezes		14.864	14,7	86.072	85,3	< 0,001	89.614	88,8	11.322	11,2	< 0,001
	Quase nunca/nunca		5.114	41,0	7.359	59,0		8.046	64,5	4.427	35,5	
Sente-se respeitado(a) pelos profissionais em relação aos hábitos culturais, costumes, religião		113.777										
	Sempre/na maioria das vezes		17.905	16,3	92.057	83,7	< 0,001	96.133	87,4	13.829	12,6	< 0,001
	Quase nunca/nunca		2079	54,5	1.736	45,5		1.896	49,7	1.919	50,3	
Tempo de consulta com o médico é suficiente		112.372										
	Sim		11.864	12,7	81.275	87,3	<0,001	83.616	89,8	9.523	10,2	< 0,001
	Não		7.854	40,8	11.379	59,2		13.230	68,8	6.003	31,2	
Tempo de consulta com o enfermeiro é suficiente		103.789										
	Sim		13.525	14,3	81.281	85,7	< 0,001	84.159	88,8	10.647	11,2	< 0,001
	Não		4.082	45,4	4.901	54,6		5.689	63,3	3.294	36,7	
Tem facilidade para falar com os profissionais que lhe atenderam para tirar dúvidas após consultas		79.216										
	Sempre/na maioria das vezes		8.062	12,3	57.404	87,7	< 0,001	59.452	90,8	6.014	9,2	< 0,001
	Quase nunca/nunca		6.073	44,2	7.677	55,8		8.766	63,8	4.984	36,2	
Encontra facilidade para conversar com os profissionais sobre os resultados dos exames		105.586										
	Sempre/na maioria das vezes		11.089	13,0	74.363	87,0	< 0,001	76.967	90,1	8.485	9,9	< 0,001
	Quase nunca/nunca		7.424	36,9	12.710	63,1		14.022	69,6	6.112	30,4	
Qualidade do cuidado recebido pela equipe de saúde		114.378										
	Muito boa/boa		9.381	10,0	84.876	90,0	< 0,001	87.284	92,6	6.973	7,4	< 0,001
	Regular/ruim/muito ruim		10.780	53,6	9.341	46,4		11.205	55,7	8.916	44,3	

Com relação à associação entre a infraestrutura e satisfação dos usuários (Tabela 4), evidenciou-se que os indivíduos que recomendariam o serviço de saúde a um amigo ou familiar e que não mudariam de equipe ou serviço foram aqueles que referiram: boas condições de uso e limpeza das instalações do serviço de saúde (p < 0,001); quantidade suficiente de cadeiras para as pessoas sentarem no local de espera (p < 0,001); muito boa ou boa percepção sobre as instalações do serviço de saúde (p < 0,001).

**Tabela 4:** Satisfação de usuários em relação à infraestrutura de serviços de atenção primária à saúde. Brasil, 2014 (n = 114.615).

		n total	Se tivesse a opção, mudaria de equipe ou serviço de saúde					Recomendaria o serviço de saúde para um amigo ou familiar				
			Sim		Não		p	Sim		Não		p
			n	%	n	%		n	%	n	%	
As instalações do serviço de saúde estão em boas condições de uso		114.615										
	Sim		10.164	11,9	75.038	88,1	< 0,001	77.241	90,7	7.961	9,3	< 0,001
	Não		10.072	34,2	19.341	65,8		21.376	72,7	8.037	27,3	
As instalações do serviço de saúde estão em boas condições de limpeza		114.615										
	Sim		13.526	13,8	84.460	86,2	< 0,001	87.562	89,4	10.424	10,6	< 0,001
	Não		6710	40,4	9.919	59,6		11.055	66,5	5.574	33,5	
Quantidade suficiente de cadeiras para as pessoas sentarem no local de espera		114.615										
	Sim		6265	10,2	55.390	89,8	< 0,001	56.449	91,6	5.206	8,4	< 0,001
	Não		13971	26,4	38.989	73,6		42.168	79,6	10.792	20,4	
Percepção sobre as instalações do serviço de saúde		114.071										
	Muito boa/boa		6278	8,6	66.958	91,4	< 0,001	67.891	92,7	5.345	7,3	< 0,001
	Regular/ruim/muito ruim		13860	33,9	26.975	66,1		30.297	74,2	10.538	25,8	

## DISCUSSÃO

Importante achado do presente estudo é que a maioria dos usuários entrevistados referiu satisfação em relação ao acesso, à infraestrutura e à qualidade dos serviços na APS no Brasil.

A distância entre o serviço de saúde e o domicílio desempenhou um significativo papel na satisfação, corroborando com os resultados da avaliação do 1º Ciclo do PMAQ-AB^16^. A satisfação pode estar relacionada com a possibilidade de não usar meio de transporte para chegar ao serviço e com o curto tempo necessário para realizar o percurso^17^. Análises de dados do PMAQ-AB de 2012 em Pernambuco evidenciaram que a maioria dos usuários (73,2%) considerava que os serviços de saúde se encontravam próximos de seus domicílios, algo já esperado tendo em vista que os serviços de saúde com menor complexidade costumam estar mais próximos da população^18^. Entretanto, a simples proximidade, por mais que seja um aspecto importante a influenciar na satisfação dos usuários, não garante a real utilização.

Em relação à satisfação dos usuários quanto ao horário de funcionamento, diversos trabalhos realizados no âmbito da APS, corroborando com o presente estudo, encontraram resultados positivos^17^^–^^19^. O funcionamento do serviço de APS em no mínimo cinco dias na semana, com carga horária de, pelo menos, oito horas, com disponibilidade de medicamentos e resultados de exames, bem como consultas com especialistas asseguradas, além de maior quantidade de unidades de saúde no município, foram identificados como fatores que tornam o serviço mais acessível e, dessa forma, satisfatório para os usuários^17^^–^^19^.

Entretanto, os resultados da pesquisa sobre o 1º ciclo, realizado entre 2012 e 2013, evidenciaram que o funcionamento da APS, apenas em horário comercial, não atende às necessidades da população entrevistada nas cinco grandes regiões brasileiras^16^. O fechamento dos serviços de APS por determinado período para a realização de reuniões das equipes, preconizado como essencial pelas diretrizes nacionais para o trabalho na APS, contribui para a insatisfação de usuários com relação ao horário de funcionamento dos serviços^17^.

Outro item importante de satisfação dos usuários em relação ao acesso é o atendimento à demanda espontânea. Observa-se que, entre os ciclos 1 e 2 do PMAQ-AB, as reservas de vagas para atendimento no mesmo dia passaram de 64,9% para 88,1%, sinalizando melhora neste indicador^20^. Dos usuários que procuram os serviços de APS para algum atendimento imediato, a maioria relata ter conseguido a consulta^19^.

Os resultados encontrados referentes à facilidade de acesso aos serviços pela maioria dos usuários entrevistados coincidem com o encontrado na avaliação do 1º ciclo do PMAQ-AB, na qual 83% das equipes da APS foram classificadas como acessíveis para os usuários^19^. Condições técnico-assistencial, econômica, política e simbólica são constituintes do conceito de acesso aos serviços de saúde^7^, entretanto, essas condições podem ser afetadas por questões como localização, população e características socioeconômicas, que podem influenciar o acesso ao serviço de APS^21^. Alguns atributos da APS demonstraram melhora entre os ciclos 1 e 2 do PMAQ, como a integralidade e o primeiro contato^20^. Tais atributos podem ter influenciado o nível de satisfação dos usuários com o serviço e colaborado para redução nas desigualdades no acesso e utilização dos serviços de saúde do Brasil^22^.

Quanto ao alto percentual de usuários que consideraram que os profissionais e os serviços de saúde se apresentam acessíveis e resolutivos às suas necessidades quando procuram os serviços de APS, esse desfecho positivo pode ter sido produzido pela fragilidade no processo de seleção dos entrevistados, visto que o mesmo só levou em consideração a opinião do grupo que conseguiu acesso ao atendimento, sendo este um dos fatores limitadores desse estudo.

As fragilidades existentes na rede de coordenação do cuidado da APS influenciam no percentual de resolutividade dos problemas de saúde dos usuários^23^. Se a correta captação da necessidade do usuário puder ser realizada e coordenada pela atenção primária, porta de entrada do Sistema Único de Saúde (SUS), o percentual de satisfação quanto ao serviço pode ser aumentado, o que os leva a indicar os serviços de APS para seus familiares e conhecidos^24^. Isso pode ser decorrente do fato de que o usuário não precisa percorrer longos caminhos na rede especializada ou alta complexidade.

Quanto à existência de consultório para atendimento com privacidade ao paciente, os resultados encontrados ratificam estudos que identificaram que a satisfação do usuário diminui 41% quando o consultório não está em uma área reservada^25^, seja para troca de roupa ou realização de exames^26^. A falta de privacidade tem uma dimensão ética, afeta a dignidade humana, dificulta a escuta, o desenvolvimento do vínculo e da confiança na relação entre usuário e profissional. Esses aspectos têm relação com o tempo que o profissional médico ou enfermeiro despende com o paciente, que também tem associação significativa com a satisfação dos usuários dos serviços^1^^,^^27^^,^^28^.

Em relação ao respeito aos hábitos culturais, costumes e religião, os indivíduos que se sentem respeitados pelos profissionais têm forte influência na avaliação positiva dos serviços de saúde^1^^,^^25^, como evidenciado pelo presente estudo e pelo de Protasio et al.^25^ Esse aspecto de satisfação pode estar associado aos modos de produção do cuidado, que quando orientados pela “valorização dos saberes, da experiência e da autonomia dos usuários, pelo entendimento do outro como ser semelhante, tendem a contribuir para ações mais criativas e que qualificam o cuidado na APS”^29^.

Em relação à infraestrutura dos serviços de APS, houve predominância de avaliação positiva das instalações pelos usuários. Condições políticas, organizacionais e estruturais que garantem a execução de funções essenciais das Unidades de Saúde é condição para maior satisfação dos usuários e desafio para construção de uma APS forte e abrangente^30^.

O estudo demonstrou que a satisfação dos usuários está associada a aspectos elementares para funcionamento dos serviços de APS, que dependem do compromisso dos gestores para com os espaços de realização do cuidado, que devem ter boas instalações físicas, condições salubres de higiene e limpeza, mobiliário, materiais e insumos suficientes para o exercício das práticas profissionais.

A redução da satisfação dos usuários às dimensões do acesso, qualidade do atendimento e infraestrutura dos serviços, assim como a dificuldade em conceituar o termo qualidade na avaliação de um serviço, trazem limitações ao presente estudo. Entretanto, as variáveis selecionadas para avaliar a satisfação permitiram traçar um panorama de como os usuários percebem os serviços da APS. A utilização de dados secundários, de uma forma geral, poderia se constituir numa limitação ao estudo, entretanto, os dados secundários utilizados no presente estudo são de um banco de dados público proveniente de uma pesquisa nacional a qual seguiu os critérios de padronização de coleta a fim de proporcionar qualidade dos registros.

Os elementos de satisfação dos usuários em relação ao acesso, qualidade e infraestrutura dos serviços de saúde ratificam a importância de se garantir, cada vez mais, que as pessoas tenham condições não só de acessar os serviços de saúde, como também sejam atendidas em locais com estruturas físicas adequadas e com profissionais capacitados a lidar com as diferenças e singularidades.

Os usuários recomendariam e não mudariam de equipe se o tempo de deslocamento ao serviço fosse curto, houvesse facilidade para acessá-lo, bom horário de funcionamento, qualidade do atendimento à demanda espontânea, resolução dos problemas no próprio serviço, existência de orientações em saúde, respeito às diversidades, tempo suficiente de consulta, facilidade para sanar dúvidas diversas e sobre exames, qualidade do cuidado recebido, e boa infraestrutura.

Nessa perspectiva, para a garantia da melhoria da qualidade dos serviços ofertados pelos serviços de APS no Brasil, os aspectos de satisfação dos usuários identificados no presente estudo devem ser considerados na organização e gestão dos serviços.
